# The Trajectory of Depression through Disenfranchised Grief in Young Widows in Times of COVID-19: A Case Report from Rural India

**DOI:** 10.3390/bs13080653

**Published:** 2023-08-04

**Authors:** Shagufta Nasir, Lydia Giménez-Llort

**Affiliations:** 1 Department of Psychiatry and Forensic Medicine, School of Medicine, Universitat Autònoma de Barcelona, 08193 Barcelona, Spain; shagufta.shagufta@autonoma.cat; 2 Institut de Neurociències, Universitat Autònoma de Barcelona, 08193 Barcelona, Spain

**Keywords:** disenfranchised grief, widowhood, rural India, COVID-19 pandemic, bioecological model, coping

## Abstract

The COVID-19 pandemic was one of this century’s deadliest and most widespread viral outbreaks, with higher mortality rates in men than women. Disruptions in funeral rituals and customs, no social recognition of the losses, and limited social support have complicated the grieving process and are linked to disenfranchised (not openly acknowledged, socially recognized, or publicly mourned) grief. Depression is also highly comorbid with complicated grief. Losing a spouse can be devastating, and this is more severe for women with limited or no resources, who are vulnerable because of the patriarchal society. In the current COVID-19 era, increased uncertainty and disenfranchised grief can worsen the clinical scenario and hamper interventions, as highlighted by the present case report on disenfranchised grief with depressive symptoms in a 30-year-old woman from rural India who, after a year of marriage, lost her husband due to COVID-19. This case study emphasizes the impact of multiple types of disadvantages due to sociodemographic and cultural determinants that can complicate the grieving process in the current context. The bioecological model of grief recovery considers individual features and societal/environmental factors to postulate the appropriate intervention. Finding meaning and purpose in life and restoration-oriented coping were successful for the clinical management of the patient.

## 1. Introduction

Spousal bereavement is considered one of the most distressing and stressful events [1]. Experiencing an adverse event like the loss of a spouse can trigger depressive symptoms [2]. The common symptoms are sadness, loneliness, diminished appetite, disturbed sleep, and frequent crying spells. A meta-analysis study estimated that 41% of adults reported clinical levels of depressive symptoms, and 27% of them reported significant anxiety levels in widowhood [3]. Widowhood could be the most disturbing experience in an individual’s life, linked to many adverse mental and physical health effects, especially if a spouse is lost in the early years of marriage [4]. According to Trivedi et al. [5], younger widows are less emotionally prepared to cope and deal with the loss of their spouses as compared to older widows. The condition of groups who are socio-economically disadvantaged, illiterate, rural residents, and from an underprivileged caste could be more detrimental.

Pre-existing inequalities during the COVID-19 pandemic aggravated the risk of mental health issues among women from disadvantaged populations. The mortality rates from COVID-19 were 77% higher in men than women, especially among middle-aged males [6]. A study by Kushwaha et al. [7] highlighted that the infection and mortality rates were higher in men than in women in India. The higher number of COVID-19-dependent deaths for males resulted in millions of female widows. It has been reported that COVID-19 widows may experience a more elevated risk of mental health conditions and physical illnesses compared to pre-pandemic widows [8].

In Indian society, especially in rural backgrounds where widows are generally a disenfranchised group [9], widowhood is not only a personal status but also a social institution. Widows often face discrimination, subjugation, and social stigmatization within their families and communities. Women’s responsibilities change after a spouse’s demise, and adjusting to a couple-oriented society as a single woman can aggravate emotional distress. Widowed women’s condition is worse, and widows are more vulnerable in rural regions. Indian studies have identified an association between being a widow, isolation, poor psychological well-being, and psychiatric illnesses. Widows may experience sub-clinical depression during the first few years following the loss of their spouse [5,10]. It is concerning that there is a lack of literature on mental health in rural communities. It would be valuable to conduct more research on this topic to understand better and meet the mental health needs of people living in rural areas.

During the pandemic, it was estimated that approximately 60 million people worldwide were mourning the loss of their loved ones [11]. The shared experience of loss in society has given rise to collective grief, and the grieving process has shifted from individual mourning to societal mourning [12]. Nonetheless, there were instances where people’s losses and grief went unacknowledged by society, which left them feeling unsupported and neglected, leading to disenfranchised grief. Doka (2008) [13] defined disenfranchised grief as the condition of a person who experiences a significant loss without society acknowledging it and providing support, which prevents public mourning. During the pandemic, many experienced psychic numbing, illustrating the concept that the death of one is tremendously significant, but as the number of deaths increases, our feeling of loss will not proportionately increase [14]. It can be better understood with a quote by Stalin Joseph, “The death of one man is a tragedy, a million deaths are a statistic”. Experiencing such events greatly elevates the likelihood of an individual encountering disenfranchised grief [15].

India’s situation appeared to be no different compared to that of any other country, but it has been confirmed that India reported some of the highest COVID-19 death tolls and confirmed cases in the world. In India, as in other low- and middle-income countries, COVID-19 affected all sections of society; yet, the pandemic and its aftermath have disproportionately impacted the disadvantaged members of the society, including women, older persons, individuals living in poverty, people with pre-existing physical or mental illnesses, and those living in certain geographical locations [16,17]. People living in rural areas face everyday obstacles to their livelihoods and have to confront barriers when seeking facilities for mental health care. The latest data for the year 2022 indicates that approximately 69% of the Indian population resides in rural areas [18]. One of the largest demographics in the nation experiences impediments in receiving mental health care.

This present case study outlines the psychosocial challenges experienced by a young widow living in a rural environment. This case study endeavors to evaluate spousal bereavement and to comprehend the role of secondary losses in inducing mental health conditions in young women living in rural India. The psychological conceptualization of this case and its management will be discussed.

## 2. Case Report

Geeta (pseudonym) is a 30-year-old female, living in a small village in Rajasthan, India, with her parents and siblings after the death of her husband. She presented with complaints of significant conflicts in interpersonal relationships for the last six months. In a clinical interview, she reported low mood, irritability, excessive crying, anhedonia, social withdrawal, dizziness, and excessive worry about the future, with associated disturbances in sleep and appetite for the past year. The symptoms insidiously started with the demise of her husband the previous year after one year of marriage. Geeta was in a content marriage but because of the sudden demise of her husband, she had to move to her maternal family. She reported being blamed for her husband’s death and often mistreated by her family in-law. While living with her parents, she found herself financially dependent on her family for necessities and obliged to ask her kin members for all little consumptions.

According to Geeta, she felt lonely and neglected at home and often spent most of her time alone. In her family, she is the fourth of nine siblings, of which three elder sisters are married and living with their husbands. She often felt like missing what her sisters have and feels a hollowness in her life. Moreover, her relationship with her younger siblings is not satisfying, and their conversations mostly end up with verbal quarrels. She also stated that discussions about her second marriage and mental health issues make her irritable most of the time and cause dramatic arguments with her family members.

During the sessions, she often reported being lonely and expressed the desire for a companion in her life. At this moment, she said to feel ready to have a partner, yet to be afraid of going again through the same process in her life. She feels to be physically attractive and that she could have marriage proposals, even if she has a widow status. Regarding her physical appearance, her siblings constantly tell her how sick she looks, and she feels annoyed and irritable. She also stated that she was rejected for marriage because of her widow status. Furthermore, the societal unrealistic beauty standards make her feel inadequate and lower her self-esteem.

She reported that she is making an effort to forget about her spouse every day as a coping mechanism to deal with the overwhelming pain and sadness but she has failed to do so. This has made her feel helpless and hopeless about her future. She often expressed how meaningless her world has seemed suddenly, and to have no purpose left in her life. The symptoms lasted for more than a year, but the family members only approached mental health professionals (MHPs) due to interpersonal conflicts at home.

In the initial stages of the illness, the family members failed to recognize the symptoms because of a lack of understanding, and when they became aware of them, there was no psychiatric hospital in the vicinity. To receive a consultation from a super specialty or a psychiatric hospital, they must travel 150 km. Geeta and her family members acknowledged their reluctance to seek mental health treatment earlier because of a lack of awareness about psychiatric illnesses, a shortage of mental health facilities, limited financial resources, expensive transportation, and the stigma associated with mental illness.

In her personal history, she earned a master’s degree in Hindi language and is currently preparing herself for public service examinations. Geeta feels discouraged by her family to prepare for higher education and public serving jobs. She is not sexually active and lost interest in sex completely. However, she feels aroused when she thinks about her deceased husband.

The psychiatrist prescribed tetracyclic antidepressants and benzodiazepines for a month, and she reported a slight improvement in sleep and appetite. Her medical history did not reveal any past psychiatric and medical illnesses. Her physical examinations reported blood pressure values of 130/90 mm Hg and an RR value of 20 per min, with no abnormalities detected in ECG and routine blood chemistry.

During the mental status examination, she appeared as a thin-built female who was looking very tired, dressed appropriately for the weather and the context. She was crying throughout the session and had limited facial expressions. Her psychomotor activity was decreased, with a depressed mood and restricted affect. Her thought content was dominated by depressive themes and issues related to interpersonal conflicts, but she denied any suicidal ideation. Her cognitive functions were intact.

### Psychiatric Screening

The patient met the criteria of a depressive episode but not an anxiety disorder based on the International Classification of Diseases (ICD-11). A few tests and self-report measures were administered to evaluate the signs and symptoms of her condition. On the Beck Depression Inventory, she scored 30, which indicated moderate depression; on the Beck Anxiety Inventory (BAI), she scored 19, indicating mild anxiety. The score of MMSE was not indicative of any cognitive problems. On the Inventory of Complicated Grief, she scored 38 points, indicating a significant impairment in social, general, mental, and physical health functioning and bodily pain. Some other psychological measures were used to assess the purpose of life and social support of the patient. On the Meaning in Life Questionnaire, she scored 15 points for the presence of meaning and 16 points for the search for meaning, which indicated that there were no value and purpose in her life and that she might not be very optimistic about the future.

**Figure 1:**
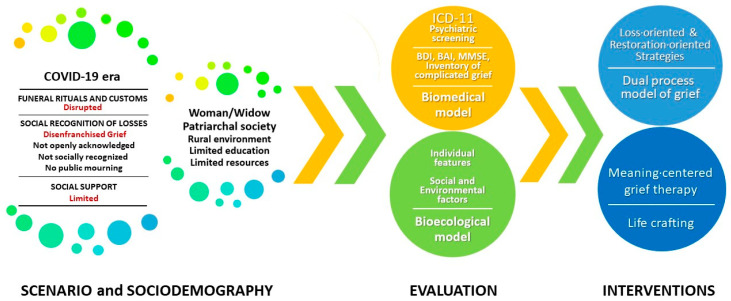
Integrative Map of the COVID-19 scenario and the sociodemographic determinants prior to biomedical-bioecological evaluation and consequent interventions.

Psychological and social difficulties are a frequent occurrence amongst women who have lost their husbands, and the situation is further exacerbated for women living in rural India because of socio-economic issues. We considered these obstacles while planning the right clinical management of the patient. Following a thorough psychiatric evaluation, a clear plan was formulated to manage her psychotherapy sessions on a bi-weekly basis. Specific strategies were implemented to support her in discovering purpose and fulfilment in her life, while also focusing on restoration-oriented coping mechanisms. These strategies were carefully designed to assist her in overcoming the challenges presented by the COVID-19 (see Figure 1).

## 3. Discussion

### 3.1. Grief and Mourning

Grief is a common response to loss; it is a non-linear, complex, and unique reaction that has personal, social, and cultural relevance [19,20] Most people adapt to the death of a loved one naturally, though not easily, along with the accompanying changes in their life circumstances [21]. Losing a loved one is a primary loss, and the losses stemming from the primary are secondary losses. Secondary losses are more prominent in all death-related losses [22]. Initially, individuals deal with the external aspect of their grief associated with performing rituals and customs, but later they deal with the internal aspect of their grief and with how they feel [23]. Alongside deaths, non-death losses during the pandemic have stricken and made the former more complex. With a loss of a closed one to death, non-death losses and secondary losses are considered too minor to be grieved [24]. These losses have been unobserved altogether and considered as disenfranchised.

When someone dies, the pattern of thoughts and routines built up for years and the assumptions about the self and the world change; these transitions are called Psycho-Social Transitions (PST) [25]. In the present case, Geeta was experiencing these transitions on superficial as well as on deeper levels (e.g., “I am no longer married now” and “I have no one to look after or forward to”). These alterations could be complex in the grieving process, which could lead to considerable levels of Prolonged Grief Disorder as well as depression, anxiety, and PTSD. They could also cause more difficulties for people who are vulnerable to anxiety and have a depressive mood/pre-existing symptoms of psychological disorders [25].

### 3.2. Death and Mourning Process during the Pandemic

The pandemic has been devastating and has a lasting influence on many lives globally. Many countries imposed national lockdowns and implemented strict movement limitations to curb the spread of the infection. The COVID-19 pandemic and the prolonged lockdown portrayed a gruesome and terrifying picture of the future and brought unprecedented uncertainty regarding physical and mental health, financial security, cognitive ambiguity, and social relationships. Although uncertainty is a natural and inevitable part of human lives, it is a persistent and predominant state during ongoing health crises [26]. It is not wise to quote that uncertainty is directly associated with psychological distress and mental illnesses. Moreover, intolerance of uncertainty affects the individual’s capacity to handle uncertain situations and stressors effectively [27]. At the pandemic’s beginning, uncertainty about the future was considered a known risk for developing severe emotional responses, anxiety, and stress-related symptoms in individuals [28]. This situation was further exacerbated by the growing number of active cases and death rates globally [29].

It is unfortunate that even as the mortality rates were decreasing, we continued to hear about several deaths each day. It is a reminder that we still have a long way to go in ensuring public health and safety. The loss or the death of a loved one could be the most devastating experience in a person’s life. COVID-19 deaths were sudden deaths, associated with intensive care treatment, social isolation, lack of opportunities of performing funeral rites with religious practices, and stigma related to COVID-19 infections [30]. A few studies about the grieving process already showed that circumstances such as limited opportunities for carrying out the death rituals and receiving adequate social support, as well as increased isolation can derail the grieving process [31,32].

The loss of a loved one is usually accompanied by other losses (loss of income, loss of sense of purpose and identity, loss of companionship, loss of faith, loss of confidence, etc.) [22]. For instance, Geeta experienced the loss of her partner through death, but she also lost her home, her only source of income, and her sense of herself as a wife. In addition to this, experiencing multiple losses causes a type of grief that arises in a context of a larger loss. The losses are not limited to experiencing the death of a loved one but included the loss of the sense of predictability, protection, control, and justice [33]. In these uncanny times, multiple mourning also complicates the grieving process [34]. The pandemic has caused social and economic disruption worldwide; people have lost not only their loved ones but also their livelihoods, and disadvantaged populations are at risk of falling into extreme poverty [35]. Geeta’s situation exemplifies the profound effects of encountering an unforeseen loss at a young age, deprived of emotional safety and inadequate resources, and with societal norms that fail to validate her feelings. Furthermore, inadequate funeral practices can lead to both external and internal disenfranchised grief.

Experiencing a loss during the pandemic elicited a more severe acute grief reaction than before the pandemic, suggesting that dealing with loss may be more difficult during this ongoing health crisis. A study by Gimenez-Llort (2022) [36] explained the difficulties faced by individuals in the grieving process before and after the pandemic and highlighted how sudden and unexpected scenarios can make grief more difficult. The dual process model by Stroebe and Schut (2021) [32] could provide a better understanding of the complexity of the grieving experience and a shift from individual to societal mourning during the pandemic. The dual process model (DPM) also emphasizes meaning-making during bereavement. Finding meaning and building a new life can be challenging, yet it is considered a central part of healing in response to both death and non-death losses [37], especially in the context of the COVID-19 pandemic [38].

### 3.3. Widowhood for Young Women in Times of the COVID-19 Pandemic in India

Living as a widow for a woman could be disadvantageous in numerous ways in normal times, and, as evident in the present case, the COVID-19 pandemic has exposed widowed women to more vulnerabilities [8]. After the loss of a spouse, women experience an economic and emotional shock. After losing a partner who is also the primary breadwinner in the family, women normally experience monetary consequences. The household faces an economic burden due to losing the primary earner and elevated financial responsibilities. These financial burdens and responsibilities tend to fall on women entirely, with little or no assistance from family members [39]. In rural settings, women/widows have fewer economic resources and endowment as compared to their male counterparts, which makes them more vulnerable to economic consequences and put them at a high risk of mental health problems [5].

In addition, women’s higher education levels, upper-class stature, and caste affiliations are not necessarily an advantage. Women often face employment restrictions, including limited opportunities and low wages, and often, they are not allowed to leave their homes for work [40]. In this case report, Geeta’s educational level could be seen as a protective factor but her societal and familial dynamics were significantly adverse, posing considerable challenges to her well-being. The family never supported her in being financially independent. For her family, sending their daughter to earn money is a matter of indignity. Nevertheless, these are the reasons why many widows experience substantial levels of poverty as compared to women who are not widows.

It is not uncommon for young widows to be blamed for their husband’s death. They are also shamed and stigmatized for their widowhood status. The level of social stigmatization can also differ from culture to culture; however, it was found that significant differences in challenges and circumstances exist within rural areas [41]. Widows may also be restricted from attending social and religious gatherings. Correspondingly, remarriages are also not so common; even if remarriage happens, the impediments are quite evident, including the demands of a dowry, a bride price, and more. These psycho-social issues are often associated with psychological impacts and could trigger psychiatric illnesses [10].

### 3.4. Psychotherapeutic Approaches

The psychotherapeutic approaches could be effective in dealing with complicated grieving experiences and associated psychiatric illnesses. Many conventional approaches to treat grief have been considered beneficial to the grievers, such as Interpersonal Therapy, Supportive Psychotherapy, Cognitive Behavioral Therapy, and Bereavement support groups [42]. In addition, many modified interventions are designed to help individuals deal with their grieving process. The literature suggests other two approaches that seem really promising to help individuals to deal with death or non-death disenfranchised grief during COVID-19 times. The dual process model proposes that an individual should be engaged in oscillating between a *loss-oriented approach*, whose focus is on acknowledging and addressing the emotion related to grief, and a *restoration-oriented* approach, whose focus is on making adjustments and attending to the life changes [32]. In a recent study on widows, it was found that women possibly gain benefits with restoration-oriented interventions compared to their male counterparts [43]. The second approach is the meaning-centred *grief therapy*, where a sense of meaning and purpose plays a crucial role in dealing with the loss of a loved one. This approach helps to recognize meaningful experiences and live with grieving experiences, cherishing the memories [44]. The meaning-focused coping and intervention were found advantageous in the time of the COVID-19 outbreak [45,46,47]

In Geeta’s case, both the dual process model and the meaning-centered grief therapy were considered to be beneficial. Utilizing the loss-oriented dual process model is imperative to effectively manage the multitude of emotions that surface following the demise of a spouse, for instance, those linked to factors such as rumination, remembering details of the situation leading to the death of the loved one, mourning over the loss, and both favorable and unfavorable interactions with the lost spouse. Using a restoration-oriented approach, Geeta could receive assistance in coping with social isolation and mastering tasks that her husband used to handle, such as managing finances, grocery shopping, and traveling alone, which would help in restoring her life with her new identity.

On the other hand, Geeta could potentially benefit from engaging in meaning-centered grief therapy that might prove beneficial in aiding Geeta in her journey towards reconciling with her grief, while simultaneously paying attention to her passions and aspirations. The utmost priority would be life crafting, in terms of implementing strategic planning for both the present and the future across a range of facets, including social life, career, and leisure time, with a strong emphasis on establishing and attaining objectives [48].

### 3.5. Challenges in Rural or Remote Areas of India and the Bioecological Model

Living in rural areas brought two major setbacks for Geeta. Firstly, being labeled as a widow imposed many restrictions and traditional practices on her life, along with limited access to resources. Secondly, the lack of awareness of mental health conditions and the unavailability of mental health facilities posed a challenge for both the individual and her family in seeking psychiatric assistance. As a result, her treatment was delayed, causing further complications. Despite the utility and advancements of these interventions, there may be several challenges in delivering an intervention regardless of the treatment modality in rural or remote areas. The obstacles in providing intervention could be inexhaustible; they include a lack of awareness of mental illnesses or related information among rural residents, wrong perceptions about mental health treatments, attitudinal barriers, a general hesitation in reaching out and seeking help, inaccessibility of MHPs in a town, and also unaffordability of the therapeutic sessions. The feasibility issues are realistic and could range from having to travel far for proper treatment to taking a day off from work to receive a consultation [49]. As the landscape of healthcare evolves, a few promising projects and models, such as the Atmiyata and SMART Project, are emerging and making a difference in patients’ lives. It is imperative to ensure that healthcare professionals receive adequate training to effectively incorporate the unique psychosocial and cultural differences that need to be considered for [50,51].

Mental health workers should be innovative in their approach to providing psychological support for grief and bereavement, particularly for those living in rural areas. Considering the cultural and demographical backgrounds, it is pivotal to contemplate an all-over approach to assist the patients. The *bioecological model of grief recovery* postulates the risk and protective factors preceding the loss, as well as the need to redefine and reintegrate the patients into life and help them to adapt to the changes in their everyday existence [52,53]. In the COVID-19 pandemic context, this socio-ecological analysis can be helpful to understand the severe adversities that individuals and society confront in dealing with grief’s complexity under adverse scenarios and the pressure of the chronosystem [54].

The *microsystem* is the individual’s immediate environment, including close friends, immediate family, grief counselors, workplace, peers’ groups, and involvement in religious or spiritual groups and practices. Identifying supportive factors in the immediate setting for the griever to feel safe, understood, and belonged is fundamental. Brief family psychoeducation or counseling can be planned to address the patient’s needs and protect the patient’s mental and physical health. *The mesosystem* is the interaction between two or more microsystems, including the connections between friends and family, family and religious involvement, religious groups, and people with similar experiences. Having intrapersonal interactions and connections strengthens the support to the patient. Moving forward, the exosystem focuses on the environment that does not directly impact the individual but has an indirect influence. It includes government policies, employment opportunities, and safety and security. It is imperative for MHPs to be aware of the Government (central, state, or regional) schemes and policies of pensions, job opportunities, and financial assistance for widows. These supports can help widows to manage financial burdens and may help with secondary losses. In addition, regular visits to villages and rural areas could be planned by MHPs, institutions, and organizations in the form of community outreach programs to increase the accessibility of mental health treatments. The active role of district mental health programs and the involvement of Government and Non-governmental organizations in sensitization and awareness programs are important. Community-wide strategies are requisite to be employed to enhance the awareness of the benefits people can receive from mental health treatments and timely consultations. Lastly, *the macrosystem* refers to values, traditions, and a broad socio-cultural concept. It may be difficult for the clinician to directly intervene on these aspects. However, implementing social policies and laws regarding mental health in rural or remote areas and providing healthcare, educational, and research resources will promote its social, political, and financial development [54,55].

## 4. Conclusions

This case report highlights how sudden deaths and non-death losses during the COVID-19 outbreak may obscure the grieving process. It was also found that a lack of recognition of the loss, social isolation, and physical distancing is linked to disenfranchised grief. Moreover, intricacies in the grieving process and psycho-social challenges can complicate the grieving process and put an individual at a higher risk of developing other psychiatric illnesses such as depression and anxiety.

To plan an effective treatment for grief, mental health professionals must employ community-wide therapeutic strategies while considering cultural and demographical backgrounds. The bioecological model of grief recovery can be used for rural populations, from the individual to the community level.

## Data Availability

Not applicable.
